# Stacked competitive networks for noise reduction in low-dose CT

**DOI:** 10.1371/journal.pone.0190069

**Published:** 2017-12-21

**Authors:** Wenchao Du, Hu Chen, Zhihong Wu, Huaiqiang Sun, Peixi Liao, Yi Zhang

**Affiliations:** 1 School of Computer Science, Sichuan University, Chengdu, China; 2 National Key Laboratory of Fundamental Science on Synthetic Vision, Sichuan University, Chengdu, China; 3 Department of Radiology, West China Hospital of Sichuan University, Chengdu, China; 4 Department of Scientific Research and Education, The Sixth People’s Hospital of Chengdu, Chengdu, China; Beijing University of Technology, CHINA

## Abstract

Since absorption of X-ray radiation has the possibility of inducing cancerous, genetic and other diseases to patients, researches usually attempt to reduce the radiation dose. However, reduction of the radiation dose associated with CT scans will unavoidably increase the severity of noise and artifacts, which can seriously affect diagnostic confidence. Due to the outstanding performance of deep neural networks in image processing, in this paper, we proposed a Stacked Competitive Network (SCN) approach to noise reduction, which stacks several successive Competitive Blocks (CB). The carefully handcrafted design of the competitive blocks was inspired by the idea of multi-scale processing and improvement the network’s capacity. Qualitative and quantitative evaluations demonstrate the competitive performance of the proposed method in noise suppression, structural preservation, and lesion detection.

## Introduction

With the wider application of X-ray computed tomography (CT) in both clinical intervention and diagnosis, the potential radiation dose has been a growing public concern [1]. The most encouraged guide in this field to reduce the radiation dose as low as possible while maintaining the imaging quality sufficient for diagnostic accuracy. A direct way to achieve this purpose is to lower the X-ray tube current or voltage. However, since the imaging procedure is an integration of quantum photons, insufficient photons will unavoidable generate quantum noise and degrade the quality of reconstructed images from traditional analytic method, i.e. filtered back-projection (FBP). The existing solutions can be primarily categorized into projection space filtering, iterative reconstruction and image post-processing. The approaches in all three categories aim to improve the reconstructed CT images from low-dose scans.

Projection space filtering [2] directly operates on raw projection data or log-transformed sinogram before FBP is applied. Although this kind of method has low computational cost, their results may suffer from structure distortion due to the lack of well definition image edges in projection domain. Iterative reconstruction (IR) [3] models the reconstruction problem as an objective function with prior constraints. Different priors have been proposed for dealing with the CT issues of low dose, limited angle and few views, such as nonlocal means (NLM), total variation (TV) and its variants, and sparse representation [4][5][6][7][8][9][10][11][12][13][14]. Despite promising results obtained by this type of method, their wide application is still circumscribed on account of the difficulty of accessing well-formatted raw data from commercial CT scanners and heavy computational burden. Relatively speaking, post-image processing methods can be applied directly on low-dose CT (LDCT) images and are more convenient to be combined into current CT systems. However, due to the fact that the noise and artifacts cannot be well determined in image domain, it is very hard to achieve satisfactory results by directly transplanting current general image denoising methods. In recent years, extensive attempts based on image post-processing methods have been made to deal with this problem [15][16]. Most representatively, inspired by the theory of sparse representation, Chen et al. proposed a patch-based fast dictionary learning approach to suppress both mottled noise and streak artifacts [16]. Additionally, the block-matching 3D (BM3D) algorithm has demonstrated to be efficient in several low-level image restoration tasks and was adapted to improve the quality of LDCT images [17].

Recently, deep learning has received overwhelming concerns due to its superior performance in numerous scientific research fields. In the field of medical imaging, several preliminary researches with this idea were proposed [18][19][20][21][22]. Wang et al. integrated a CNN-based sparse prior into the IR framework as the regularization term for accelerating MRI reconstruction [18]. Zhang et al. designed a simple 3-layer convolutional neural network (CNN) for limited-view tomography [19]. Chen et al. proposed a lightweight CNN method to estimate the mapping function from LDCT images to their corresponding routine-dose images [20]. Kang et al. applied the U-Net to the multiscale data of LDCT images after decomposition by the wavelet transform [21]. By combining the idea of residual encoders into traditional CNN, Chen et al. attained promising results in LDCT [22].

In spite of some preliminary results achieved by deep learning for LDCT, the power of a deeper and wider network has not been fully explored. Several studies in the field of computer vision have made considerable progress on constructing deep architecture [23][24][25]. However, to prevent from discard of meaningful structural details, most neural network models for image restoration, which is considered a low-level task, had limited the depth of network. This property is different from high-level tasks in computer vision, e.g. classification or detection [26][27], in which max-pooling operation is extensively used to capture high-level features.

**Fig 1:**
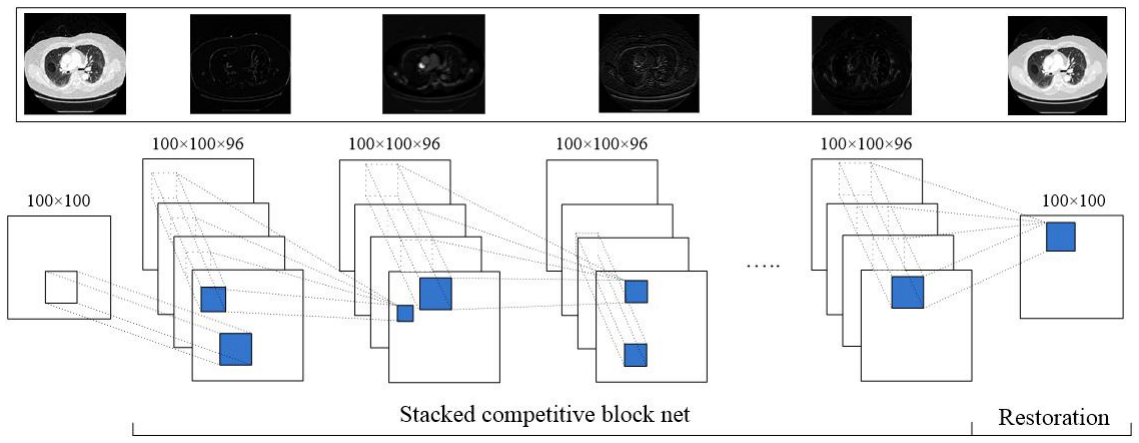
An overview of the proposed architecture SCN.

In this paper, we expand the frontier of CT analysis by adopting a deep network for processing the complex nonlinearity in LDCT. Inspired by the work of [23], we introduce a new architecture named Stacked Competitive Network (SCN), which is illustrated in Fig 1, into the traditional CNN. Instead of a single stacked CNN, our SCN is comprised of several successive Competitive Blocks (CB). Each CB introduces a multi-scale processing mechanism by increasing the width of the network, which can further improve the ability of the traditional CNN. In the second section, the proposed SCN model is elaborated. In the third section, qualitative and quantitative experiments were performed to evaluate the proposed SCN model. Finally, the conclusion was drawn.

## Method

### Single competitive block

The main idea of the single competitive block is to optimize the local structure in a CB with a certain sparsity, which can be approximately obtained by possible dense components. It is noteworthy that assuming shift-invariant means the proposed network can be constructed from stacked blocks. For this purpose, what we need to do is to identify the optimal sparse structure and repeat it spatially.

We assume that each feature map from the previous layer relates to regions of the input image, and these feature maps are clustered into different filter banks. However, single-scale filters cannot adequately capture low-level features and texture patterns simultaneously, especially in LDCT images.

Inspired by the work of [23], convolutional filters with different scales were utilized, which means that multi-scale texture and structural features could be captured in the same image region. Furthermore, because pooling layers will result in the loss of spatial information, all of the pooling operations are discarded in our blocks.

Although multi-scale filters can capture richer information of original images, they have shortcomings. First, many redundant feature maps are used repeatedly, which inevitably increases the computational burden. In addition, the introduction of a CB significantly boosts network parameters, which increases the difficult of training.

**Fig 2:**
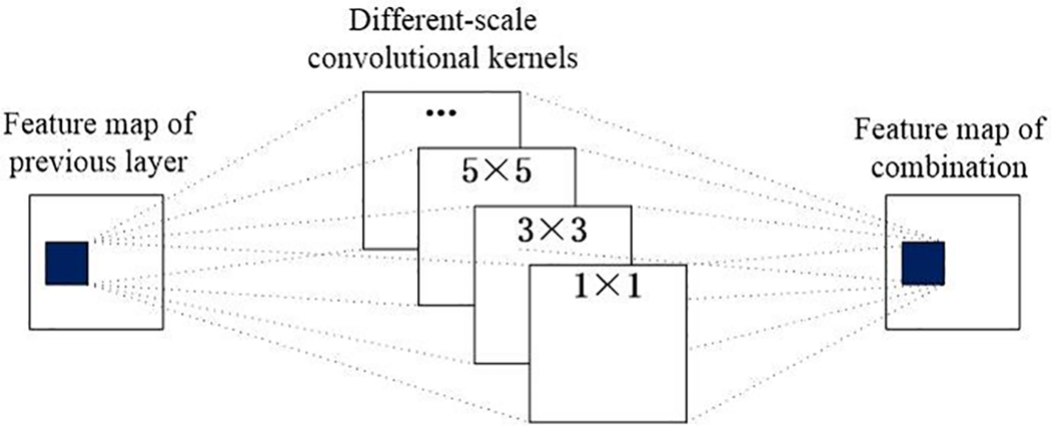
Single competitive block model.

To solve these problems, a Combination Function (CF) is designed for each block. A feature map is generated as the input of the next layer in the network. The CF can be formulated as follows:

$$a_{i}=\Phi (a_{i-1}),$$ (1)

and

$$\begin{array}{l} \Phi (a_{i-1})=\sigma (W_{i}^{1}a_{i-1}+b_{i}^{1})⊙\sigma (W_{i}^{3}a_{i-1}+b_{i}^{3})⊙\cdots \cdot \\ \cdots \cdots ⊙\sigma (W_{i}^{k}a_{i-1}+b_{i}^{k})\ldots ⊙\sigma (W_{i}^{K}a_{i-1}+b_{i}^{K}),\;k\in \{1,3,\ldots ,K\}, \end{array}$$ (2)

where *σ*(·) is the ReLU function; *a*_*i*-1_ is the feature representation of the (*i*-1)^*th*^ layer; k represents the scale kernel size of the *i*^*th*^ layer; ☉ is the element-wise max operation; Φ can be viewed as a competitive combination function, which can be implemented using the element-wise max operation in the network; and *a*_*i*_ is the new feature map constructed by CF, which is used as the input of the next layer. The structure of a single competitive block is shown in Fig 2.

### Stacked competitive networks

Since the CNN-based approaches are immune to the impact of the statistical distribution of the artifacts and noise, we model the noise reduction problem for LDCT as follows. Letting *X*∈*F*^m×*n*^ be an LDCT image, and letting *Y*_*g*_∈*F*^m×*n*^ be the corresponding normal-dose image, the noise-reduction problem can be transformed into the problem of learning a mapping function *R* directly from the LDCT to NDCT as follows:

$$R:X\to Y_{g}.$$ (3)

Generally, *R* is nonlinear and complicated. To achieve this aim, *k* single Competitive Blocks (CBs) are stacked as a deep CNN to transform the LDCT image to its corresponding NDCT image. The noise reduction problem can be formulated as the minimization of the following loss function:

$$L(\Theta )={\left\|Y_{g}(x)-\Phi_{k}(\Phi_{k-1}(\ldots \Phi_{1}(x)))\right\|}_{2}^{2},$$ (4)

where Φ_i_ is the competitive mapping function of the *i*^*th*^ layer in the proposed SCN and Θ denotes the parameters of the network. Nonlinear mapping is imposed into the first *k*−1 blocks to represent the nonlinear relationship between the LDCT and NDCT image features. The competitive block in the last layer, namely, Φ_*k*_, reconstructs the estimated NDCT image *Y*_*r*_.

To avoid over-fitting, a regularization term $\sum_{i=1}^{k}{\left\|W_{i}\right\|}_{F}^{2}$ (a weight decay term) is introduced, which forces to reduce the magnitudes of the weights. The objective function is re-formulated as follows:

$$L(\Theta )={\left\|Y_{g}(x)-\Phi_{k}(\Phi_{k-1}(\ldots \Phi_{1}(x)))\right\|}_{2}^{2}+\alpha \sum_{i=1}^{k}{\left\|W_{i}\right\|}_{F}^{2}.$$ (5)

Due to the large number of parameters, the optimization of *L* tends to fall into a local minimum. To avoid this situation, first, an unsupervised pre-training process was utilized to initialize the first *k*−1 layers in a stacked strategy, and we randomly initialize the *k*^*th*^ layer; second, we fine-tuned the whole network in a supervised way. The output of this single-hidden-layer network *a*_*i*_ = Φ_*i*_(*a*_*i*-1_) is used as the input of the next layer. The original LDCT image is used as the input of the first layer, *i*.*e*., *a*_0_ = *x*. After initialization, all of the layers were fine-turned with (Eq 5). As a result, the front layers of a stacked competitive block attempt to catch the low-level features, such as texture patterns in LDCT images, while the higher layers try to seize higher-level features that contain context information from low-level features.

Owing to the SCN is an end-to-end architecture, once the network is configured, the set of the parameters, Θ should be estimated to build the mapping function *R*. The estimation can be achieved by minimizing the loss function *L*(Θ) between the estimated CT images *X* and the reference NDCT images *Y*_*g*_. Given a set of paired patches *P* = {(*X*_1_,*Y*_1_),(*X*_2_,*Y*_2_),…,(*X*_*T*_,*Y*_*T*_)} where {*X*_*t*_} and {*Y*_*t*_} denote LDCT and NDCT image patches respectively, and *T* is the total number of training samples. In the training stage, the loss function was first optimized by Adam [28] and later by SGD [29] optimization.

## Experimental design and results

For evaluation purpose, we compared our method with five different state-of-the-art approaches: TV-POCS [10], K-SVD [16], BM3D [17], SSCN [20], and KAIST-Net [21]. K-SVD and BM3D are the two powerful image restoration algorithms, which have been successfully employed by LDCT. TV-POCS is a classical IR method constrained by a sparse gradient prior. SSCN is a first CNN-based LDCT image restoration model, which only has single-scale convolutional kernel, e.g., 5×5. We also compared to a recently presented deep CNN model, called KAIST-Net. It can be treated as an advanced variant of the SSCN model aided by multi-scale residual learning. In all the cases, the parameters in TV-POCS, KSVD and BM3D were adjusted to achieve the best result.

Three metrics, including, peak signal-to-noise ratio (PSNR), root mean square error (RMSE), and structural similarity index measure (SSIM), were chosen for quantitative assessment. All experiments were implemented in MATLAB 2017a on a PC (Inter i7-4970 CPU, 32 G RAM and GTX 980TI graphics card). The codes of this project have been released in web page (https://github.com/Wenchao-Du/SCN-for-Image-Denoising).

### Data source

#### Simulated data

**Fig 3:**
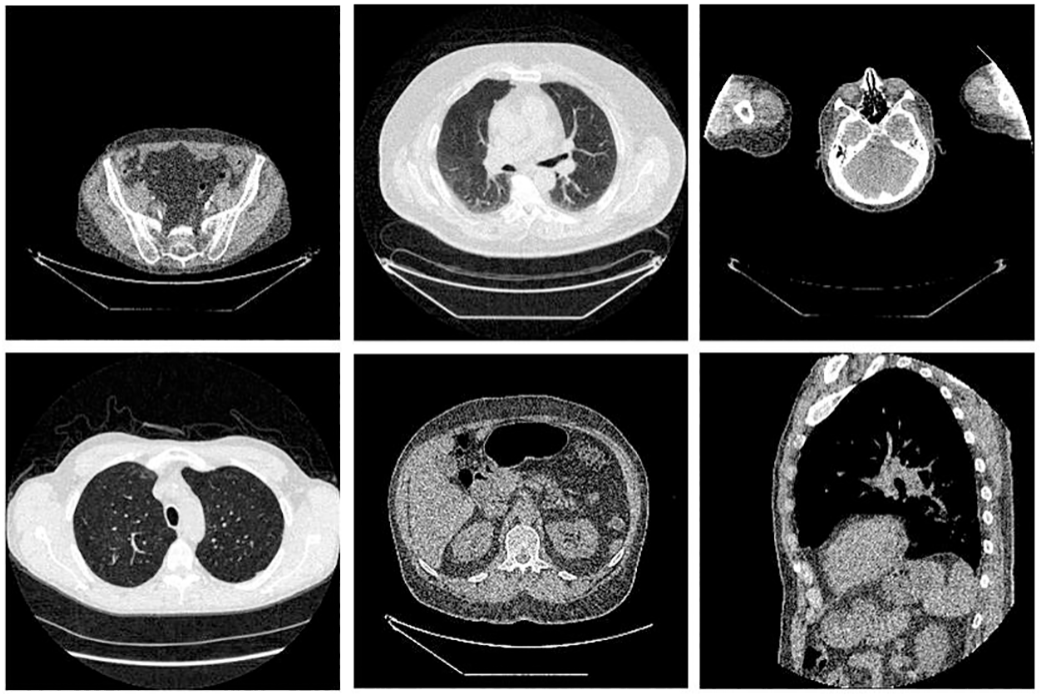
Typical LDCT images for training.

TCIA Dataset: This dataset included 7015 NDCT images from 165 patients, which were obtained from The Cancer Imaging Archive (TCIA, https://imaging.nci.nih.gov/ncia/). The image samples were 256×256 pixels. Several representative slices are shown in Fig 3. It can be seen that different body parts were involved to maintain the diversity of data source. The corresponding LDCT images were simulated by introducing Poisson noise into the projection data from the NDCT images. Under the assumption of monoenergetic source, the projection data obey the Poisson distribution that can be formulated as

$$z_{i}∼Poisson\left\{b_{i}e^{-l_{i}}+r_{i}\right\},i=1,\ldots ..,I,$$ (6)

where *z*_*i*_ is the measurement along the *i*^*th*^ X-ray path, *b*_*i*_ is the blank scan factor, *r*_*i*_ denotes the electronic noise, and *l*_*i*_ is the integral of the X-ray attenuation coefficients. In our simulations, the noise level can be easily controlled with different *b*_*i*_. In the initial experiments, *b*_*i*_ was uniformly set to 10^5^ photons and denoted as *b*_0_ = *b*_*i*_ = 10^5^, *i* = 1,… ..,*I*. The Siddon ray-driven algorithm was utilized to produce the projection data with a fan-beam geometry. The detector-to-rotation center distance was 400 mm and the rotation center-to-source distance was 400 mm. The physical size of image was 200 mm×200 mm. The length of detector was 413 mm and the detector had 512 bins. Over a 360° range, 1024 projections were uniformly sampled.

Since directly processing the complete image is knotty, SCN was applied on image patches instead. Usually, deep neural networks require amount of training samples, which will be difficult to obtain in the medical field due to the limitations of patient privacy. Extracting image patches with overlapped sliding windows is an efficient way to enlarge the training set.

In our experiments, 200 NDCT and corresponding simulated LDCT image pairs were randomly chosen for training and 100 images pairs were randomly chosen for testing. The test data is chosen from a different subject than the training set.

#### Clinical data

Mayo Clinical Dataset: This dataset included 2378 3-mm-thickness routine- and quarter-dose CT images from ten patients. The usage of Mayo dataset was provided by Low Dose CT Grand Challenge (http://www.aapm.org/GrandChallenge/LowDoseCT/). The training set composed of a portion of routine- and quarter-dose image pairs. The testing set contained the rest of the image pairs. For comparison, 10-fold cross validation was used in the testing stage: the images from nine patients were involved in the training stage and the rest one was used as testing samples.

### Parameter selection

The training data consist of pairs of image patches with size of 100×100 that were extracted from LDCT images with a sliding distance of four pixels. After extracting image patches, the number of training samples reached 10^6^. The network was implemented with Caffe. In our experiments, we evaluated several parameter combinations and finalized the parameter setting as follows. The learning rate was initialized to 10^−2^ and gradually decayed to 10^−5^. The convolutional kernels were initialized with random Gaussian distributions with zero mean and standard deviation 0.01. The numbers of filters in the layers were 96 except the last layer, which was set to 1. To avoid patch-alignment issues and heavy computational burden, the competitive blocks were restricted to 3 filters with sizes of 5×5, 3×3 and 1×1. Due to the flexibility of CNN, the proposed SCN can perform on patches with arbitrary size. All of the testing samples were fitted into the network directly without any pre-processing operations.

### Experimental results

#### Simulated data

**Fig 4:**
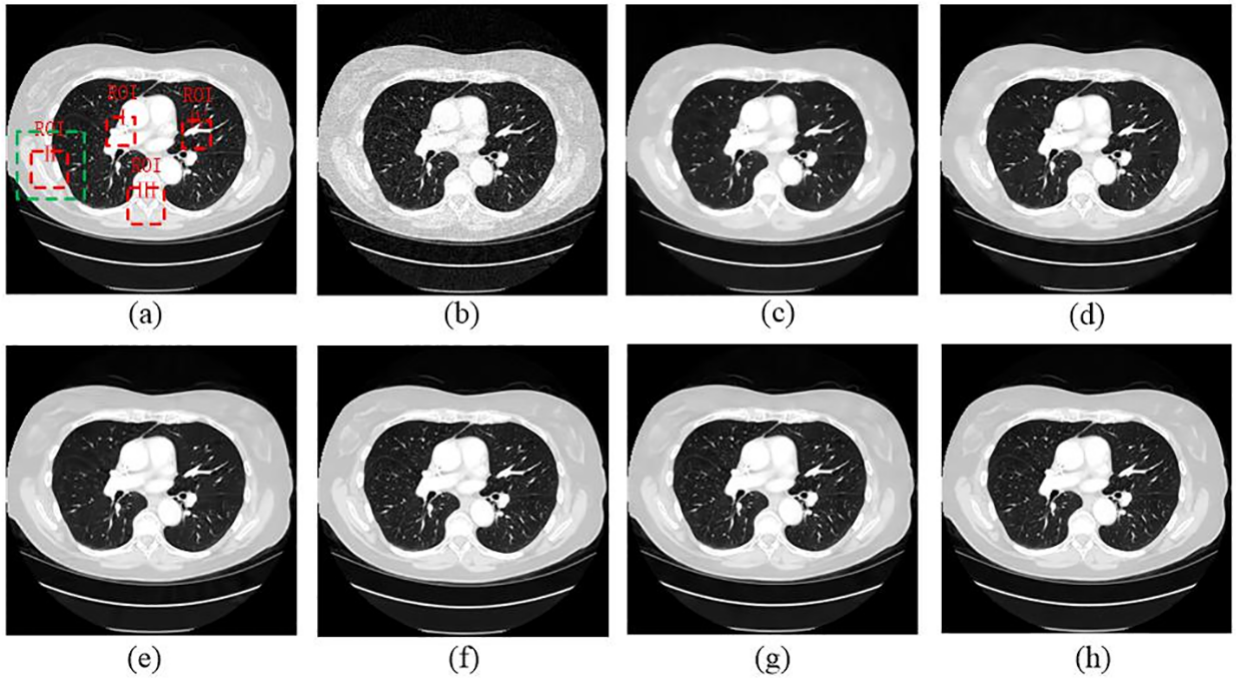
The thoracic image processed by different methods for comparison. (a) NDCT, (b) LDCT, (c) TV-POCS (*λ* = 0.08), (d) K-SVD (*σ* = 4, *n* = 80000, *block_size* = 8), (e) BM3D (*σ* = 9.5), (f) SSCN, (g) KAIST-Net, and (h) SCN. The green box denotes the region that is magnified in Fig 5. Several ROIs were defined by red rectangles.

**Fig 5:**
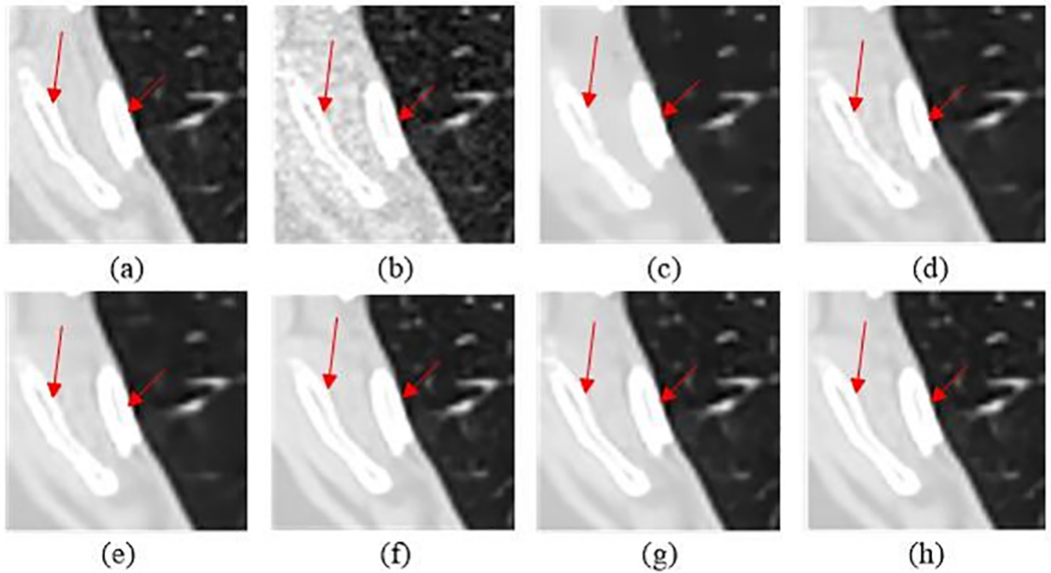
Magnified part marked by a green box in Fig 4(a). (a) NDCT, (b) LDCT, (c) TV_POCS, (d) K-SVD, (e) BM3D, (f) SSCN, (g) KAIST-Net, and (h) SCN ((a)-(h) from Fig 4(a)–4(h)). The arrows mark two locations with visible differences.

In order to evaluate the performance of the proposed SCN, two typical slices, which were from thorax and abdomen, were selected. Due the difference of scan protocols between two slices, the noise and artifacts in both images appeared in different degrees. Fig 4 demonstrates the thoracic image processed by different methods. In Fig 4(b), it is obvious that the LDCT image has severe streak artifacts and noise, especially close to the tissues with high attenuation coefficients, e.g. bones. All the approaches showed different abilities on noise and artifact reduction. In Fig 4(c), it is noticed that TV-POCS smoothed some small details in the pulmonary lobes due to its notorious blocky effect. K-SVD and BM3D had a better performance on detail preservation than TV-POCS, but the artifacts radiated from the bones were still obvious. SSCN, KAIST-Net and SCN removed most artifacts and noise and the structural information were maintained better than other methods. In addition, SCN better distinguished the low-contrast regions. Fig 5 shows the magnified the region from different methods, which was indicated by the green box. Obviously, the parts pointed by the blue arrow were blurred in Fig 5(c). The other methods can discriminate these details to various degrees. In Fig 5(h), the details were well preserved without blurring. In Fig 5(d) and 5(e), the streak artifacts can be observed adjacent to the bones, which are indicated by the red arrow.

**Fig 6:**
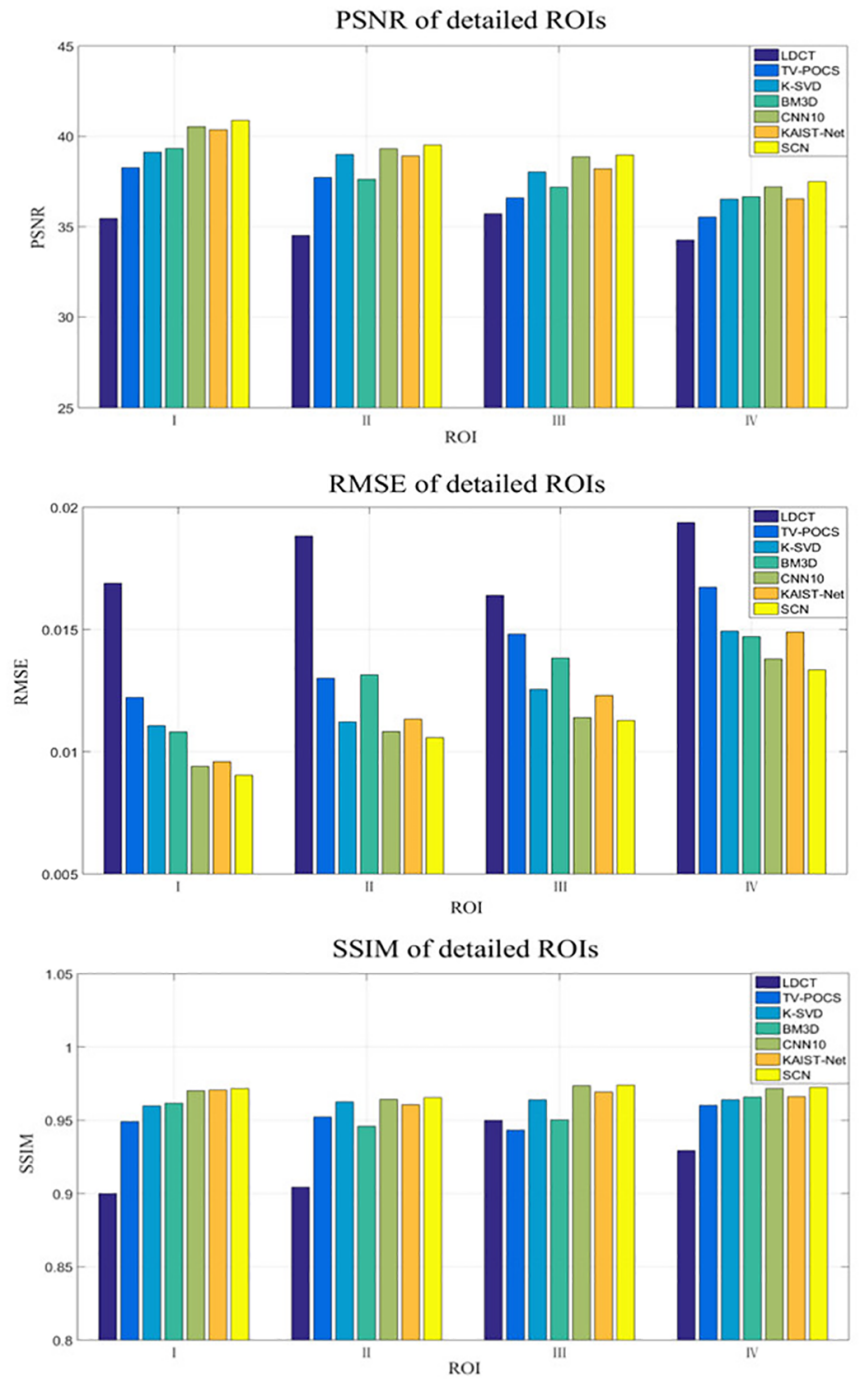
Statistical result from different methods over the ROIs indicated in Fig 4(a) in terms of the specific metrics.

Four ROIs, which are indicated by red dotted rectangles in Fig 4(a), were selected for quantitative assessment. The magnified ROIs are shown in Fig 6. It can be noticed that the quantitative results had coherent trend to visual effect. The SCN had the best values in all the metrics for all the ROIs.

**Fig 7:**
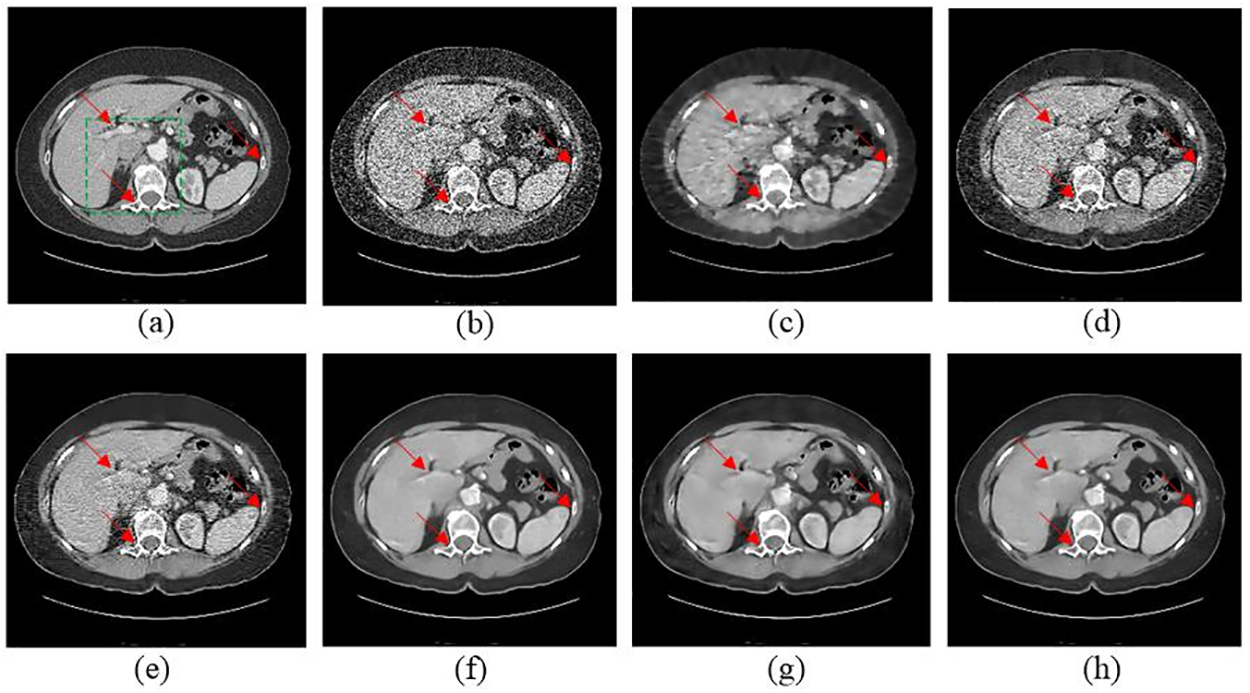
The abdominal image processed by different methods for comparison. (a) NDCT, (b) LDCT, (c) TV-POCS (*λ* = 0.08), (d) K-SVD (*σ* = 4, *n* = 80000, *block_size* = 8), (e) BM3D (*σ* = 9.5), (f) SSCN, (g) KAIST-Net, and (h) SCN. The arrows indicate three locations in which the visible differences can be observed.

**Fig 8:**
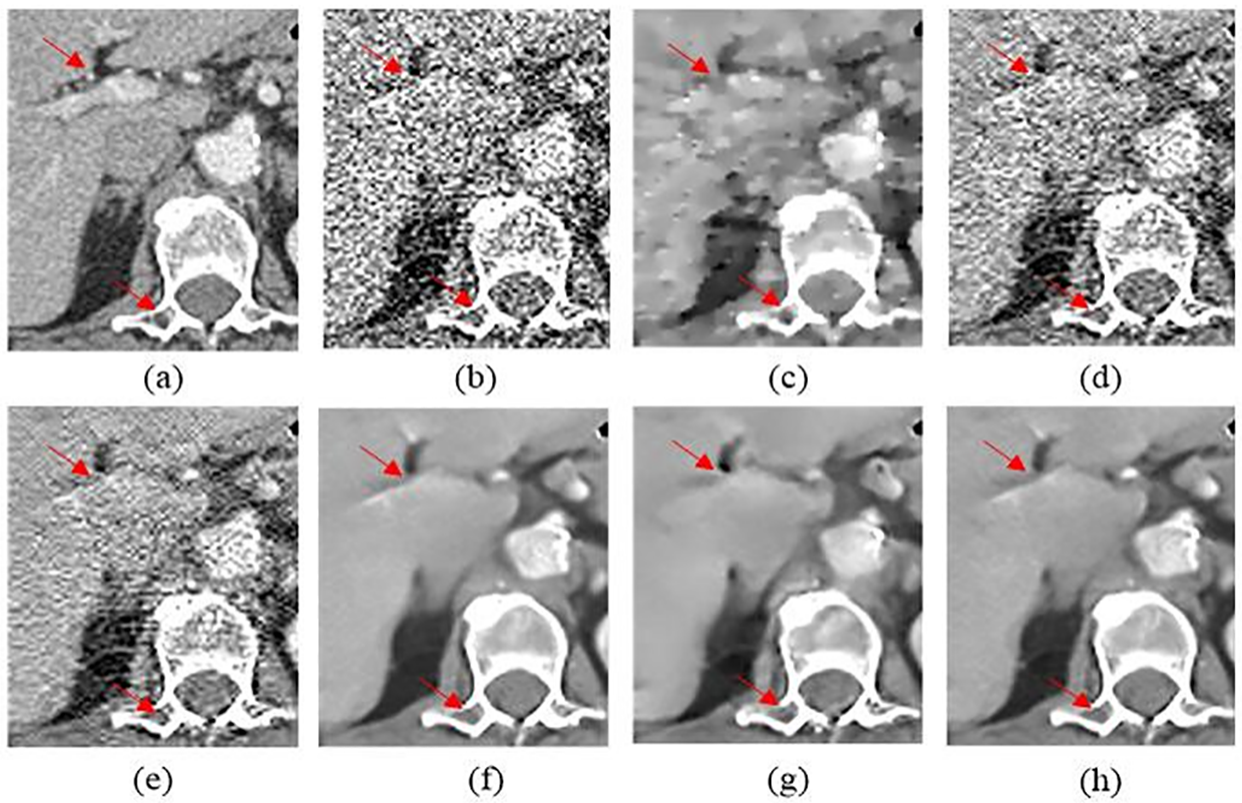
Magnified part marked by a green box in Fig 7(a). (a) NDCT, (b) LDCT, (c) TV-POCS, (d) K-SVD, (e) BM3D, (f) SSCN, (g) KAIST-Net, and (h) SCN ((a)-(h) from Fig 7(a)–7(h)). The arrows point to two locations with structural information that were recovered differently by the competing methods.

Fig 7 gives the abdominal image processed by different methods. Since the routine-dose abdominal image (Fig 7(a)) is noisier than routine-dose thoracic image (Fig 4(a)), it will be much more difficult to distinguish the structures due to the severe deterioration in low-dose abdominal image (Fig 7(b)). TV-POCS and K-SVD had limited performance in recovering the details as shown in Fig 7(c) and 7(d). The result in Fig 7(c) is still contaminated by blocky effect. Although BM3D suppressed most noise, the artifacts near the vertebral column are evident. SSCN, KAIST-Net and SCN eliminated most of the artifacts and noise, but the results in Fig 7(f) and 7(g) suffered from mild blurring and this drawback was also mentioned in our previous work [14]. Several regions with detectable structure differences are marked by the red arrows. The red arrow in the middle of the liver point to a structural detail, which was well retained by SCN in Fig 8. Other tissues, such as vertebral column and bones were best maintained in the results of SCN as well.

The quantitative results for the abdominal image processed by different approaches are shown in Table 1. It can be seen that SCN achieved an impressive result on all indices. Table 2 shows the statistical results for all the 100 images in the testing set. The proposed SCN outperformed other competing methods in all of the metrics.

**Table 1:** Quantitative results associated with different algorithms for the abdominal image.

	PSNR	RMSE	SSIM
**LDCT**	32.4435	0.0239	0.7416
**TV-POCS**	38.8888	0.0114	0.9054
**K-SVD**	36.7985	0.0145	0.8904
**BM3D**	38.7910	0.0115	0.9294
**KAIST-Net**	40.9523	0.0090	0.9572
**SSCN**	41.5981	0.0083	0.9622
**SCN**	**41.8059**	**0.0081**	**0.9625**

**Table 2:** Quantitative results (mean±std) associated with different algorithms for the images in the testing dataset.

	PSNR	RMSE	SSIM
**LDCT**	36.3975±2.5430	0.0158±0.0050	0.8635±0.0659
**TV_POCS**	41.5021±2.3344	0.0087±0.0024	0.9430±0.2241
**K-SVD**	40.8445±2.7059	0.0096±0.0036	0.9497±0.0380
**BM3D**	41.5358±2.6471	0.0088±0.0033	0.9597±0.0292
**KAIST-Net**	43.1295±2.1453	0.0072±0.0019	0.9742±0.0120
**SSCN**	43.6658±2.0257	0.0067±0.0017	0.9774±0.0123
**SCN**	**43.9120±2.0828**	**0.0066±0.0017**	**0.9777±0.0105**

#### Clinical data

**Fig 9:**
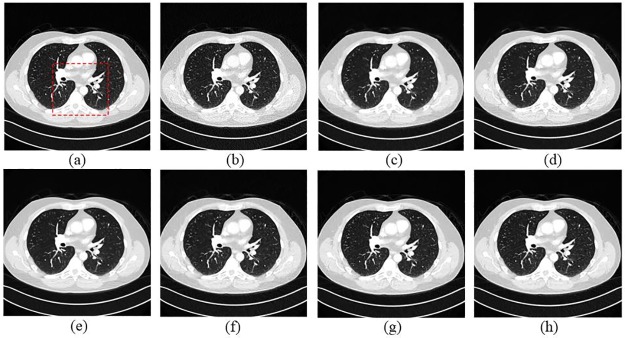
The thoracic image processed by different methods for comparison. (a) NDCT, (b) LDCT, (c) TV-POCS (*λ* = 0.08), (d) K-SVD (*σ* = 4, *n* = 80000, *block_size* = 8), (e) BM3D (*σ* = 9.5), (f) KAIST-Net, (g) SSCN, (h) SCN.

**Fig 10:**
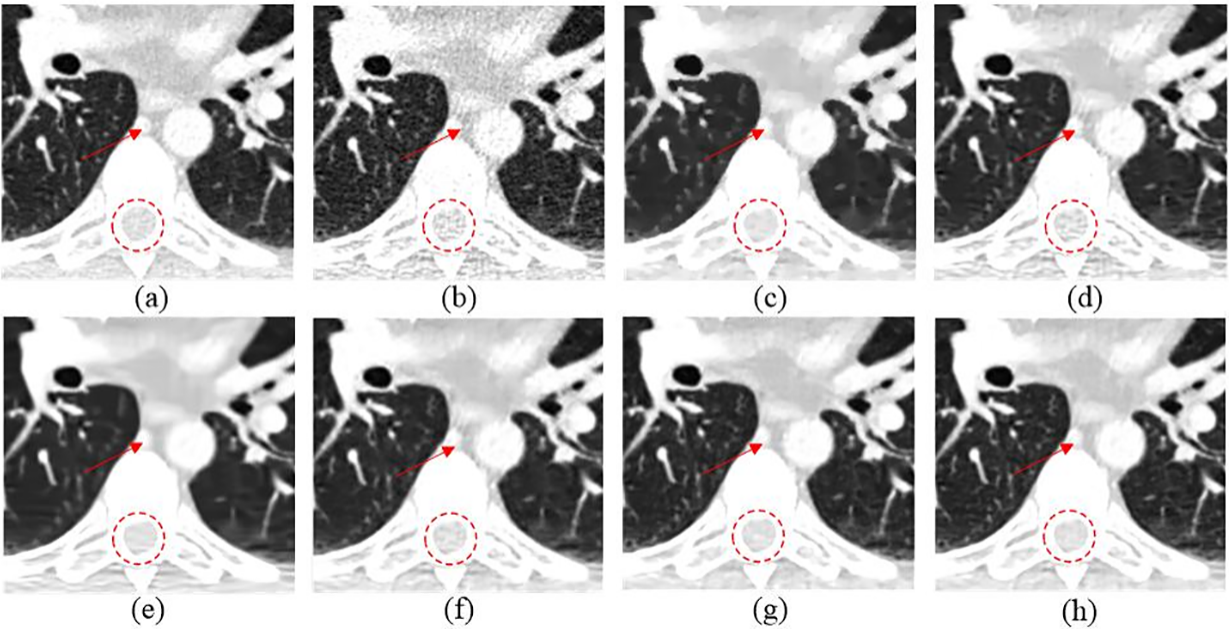
Magnified part marked by a red rectangle in Fig 9(a). (a) NDCT, (b) LDCT, (c) TV-POCS, (d) K-SVD, (e) BM3D, (f) KAIST-Net, (g) SSCN, (h) SCN. The circle denotes the spinal cord area and the red arrowhead located the contrast-enhanced intercostal vein.

To further evaluate the performance of SCN, a representative slice from Mayo clinical dataset was selected and Fig 9 shows the results. It is easy to be observed that SCN obtained the superior capability of detail reservation and noise reduction. In Fig 10, SCN yielded the best image quality for detail recovery and structure preservation, as highlighted by the red circles in the magnified parts. Extra artifacts were introduced by K-SVD and TV-POCS. BM3D over-smoothed the details. Only CNN based methods can differentiate the contrast-enhanced intercostal vein, which is indicated by the red arrowhead in Fig 10. Although KAIST-Net and SSCN achieved similar performances to SCN, the texture and structural details were better retained in Fig 10(h).

**Fig 11:**
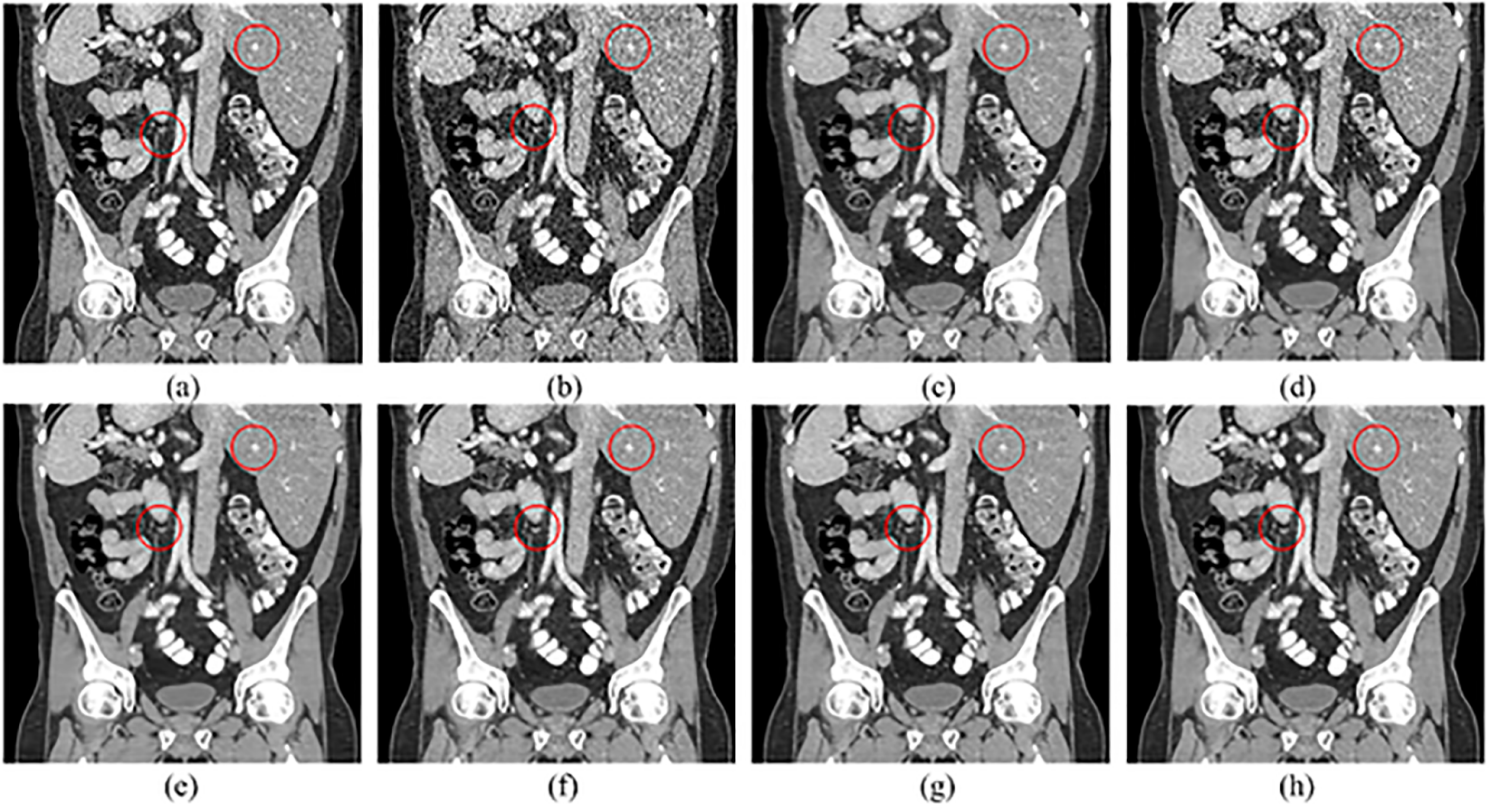
Coronal images processed by different methods for comparison. (a) NDCT, (b) LDCT, (c) TV-POCS, (d) K-SVD, (e) BM3D, (f) KAIST-Net, (g) SSCN, (h) SCN.

**Fig 12:**
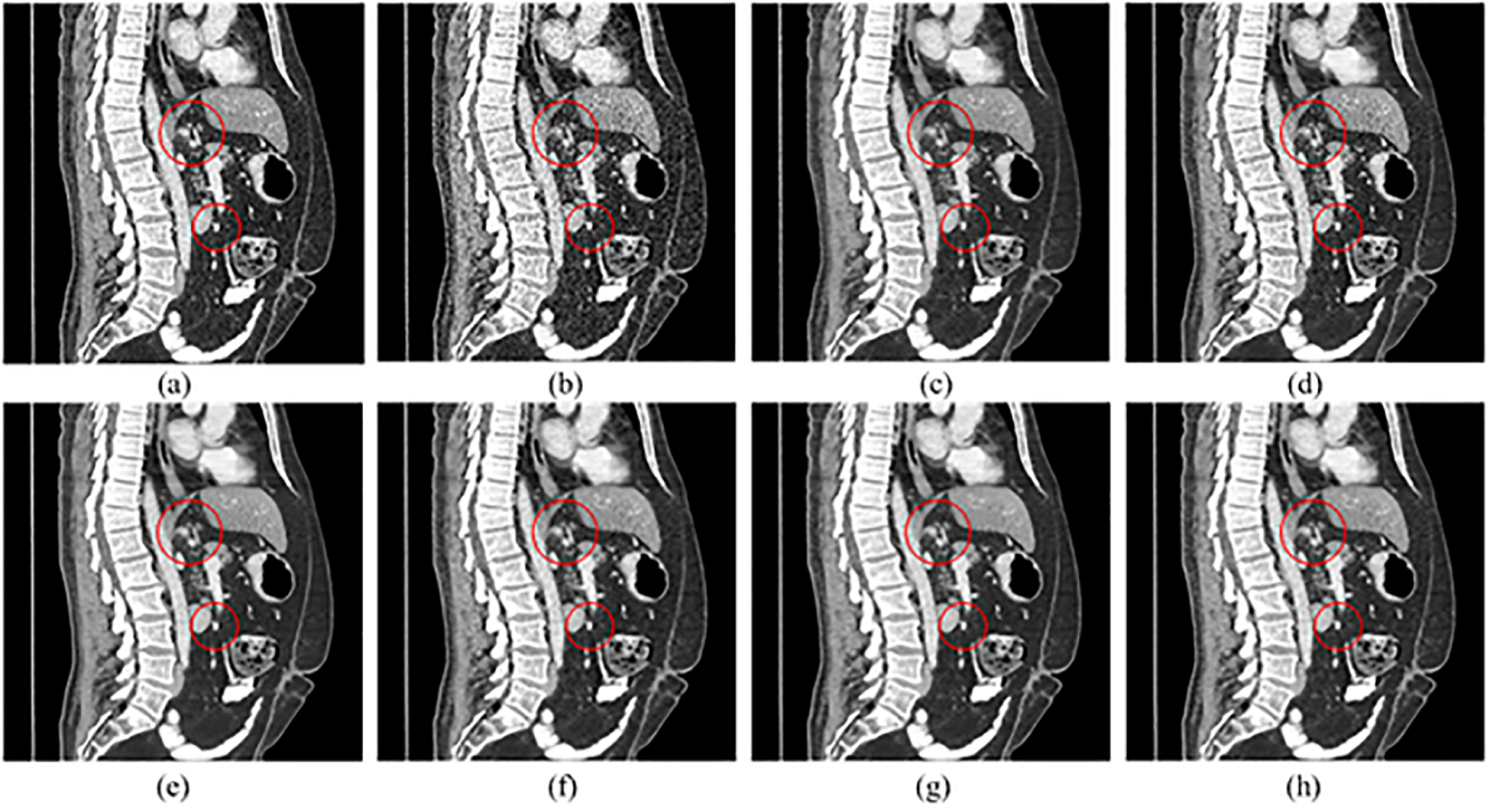
Sagittal images processed by different methods for comparison. (a) NDCT, (b) LDCT, (c) TV-POCS, (d) K-SVD, (e) BM3D, (f) KAIST-Net, (g) SSCN, (h) SCN.

The coronal and sagittal images of the results are demonstrated in Figs 11 and 12. Red circles indicate the regions with visual differences. It is clear that comparing with other methods, SCN can remove most of the noise while maintaining more structure information.

Table 3 summarizes the quantitative results of Figs 9 and 10. SCN outperformed the other methods in all the metrics. Table 4 shows the quantitative results of 10-fold cross validation in terms of MEANS±SDs.

**Table 3:** Quantitative results associated with different algorithms for Figs 9 and 10.

	Fig 9			Fig 10		
	PSNR	RMSE	SSIM	PSNR	RMSE	SSIM
**LDCT**	36.1320	0.0156	0.8646	33.6506	0.0208	0.8637
**TV-POCS**	38.3537	0.0121	0.9258	35.1303	0.0175	0.9170
**K-SVD**	39.1203	0.0111	0.9263	35.7178	0.0164	0.9212
**BM3D**	39.5289	0.0106	0.9292	36.0610	0.0157	0.9235
**KAIST-Net**	39.6314	0.0104	0.9337	36.5035	0.0150	0.9311
**SSCN**	39.9666	0.0100	0.9442	36.3006	0.0153	0.9290
**SCN**	**40.1022**	**0.0099**	**0.9443**	**36.7345**	**0.0146**	**0.9328**

**Table 4:** Quantitative results (MEANS±SDs) associated with different algorithms on cross-validation.

	PSNR	RMSE	SSIM
**LDCT**	37.5499±1.9246	0.1358±0.0030	0.8786±0.0413
**TV-POCS**	40.1523±1.6217	0.0100±0.0019	0.9372±0.0194
**K-SVD**	40.8753±2.1900	0.0093±0.0025	0.9359±0.0250
**BM3D**	41.1705±1.7350	0.0089±0.0020	0.9412±0.0177
**KAIST-Net**	41.3048±1.6877	0.0088±0.0017	0.9436±0.0180
**SSCN**	41.8338±1.8018	0.0083±0.0017	0.9524±0.0169
**SCN**	**41.9175±1.8708**	**0.0082±0.0018**	**0.9525±0.0170**

#### Investigation of competitive blocks in SCN

As the proposed SCN model composed of several successive CBs, the impact of CB was investigated. We constructed convolutional networks with the same structure but instead used the convolution operation with single-scale 5*5 and 3*3 convolutional kernels; these CNNs are denoted as CNN-5 and CNN-3, respectively. The experiments were performed on TCIA dataset.

Meanwhile, to evaluate the robustness of SCN, training and testing data with different noise levels were generated to produce the quantitative results, which are shown in Table 5. In this table, the training set of CNN-5, CNN-3 and SCN was randomly mixed with various noise levels for *b*_0_ = 10^5^, *b*_0_ = 5×10^5^ and *b*_0_ = 5×10^4^. It can be noticed that SCN still achieved the best performance, which is a powerful evidence for the merit originated from the introduction of CBs. The robustness of SCN for different noise levels is also can be confirmed.

**Table 5:** Quantitative results (mean) associated with different algorithms for combinations of noise levels.

Noise level of testing data		TV-POCS	K-SVD	BM3D	KAIST-Net	CNN-5	CNN-3	SCN
*b*_0_ = 5×10^5^	**PSNR**	44.8030	44.0576	44.2798	44.9645	45.2609	45.2998	**45.6102**
	**RMSE**	0.0061	0.0064	0.0063	0.0058	0.0056	0.0056	**0.0054**
	**SSIM**	0.9735	0.9778	0.9796	0.9827	0.9835	0.9829	**0.9837**
*b*_0_ = 1×10^5^	**PSNR**	41.5021	40.8445	41.5358	43.88781	43.392	43.4035	**43.6498**
	**RMSE**	0.0087	0.0096	0.0088	0.0075	0.0070	0.0070	**0.0068**
	**SSIM**	0.9498	0.9447	0.9509	0.9765	0.9768	0.9760	**0.9770**
*b*_0_ = 5×10^4^	**PSNR**	39.7729	38.9090	39.8928	41.8451	42.1944	42.1828	**42.4149**
	**RMSE**	0.0106	0.0121	0.0121	0.0084	0.0090	0.0080	**0.0078**
	**SSIM**	0.9221	0.9296	0.9296	0.9688	0.9714	0.9702	**0.9717**

We examined the impact of the number of convolutional kernels contained in a single CB. Several SCN networks were constructed and different numbers of convolutional kernels were included in a single CB. These networks are denoted as SCN-2, SCN-3 and SCN-4 respectively. The CBs in SCN-2 contains both 1×1 and 3×3 kernels; the CBs in SCN-3 contain 1×1, 3×3 and 5×5 kernels; the CBs of SCN-4 contains 1×1, 3×3, 5×5 and 7×7 kernels. The experiments were performed on TCIA dataset.

Table 6 shows the quantitative results. In Table 6, the training and testing sets were same as Table 5 for SCN-2, SCN-3 and SCN-4. It can be observed that SCN-4 obtained better performance, which confirmed the effectiveness of multi-scale convolutional kernels and more scales can further improve the performance. However, adding more convolutional kernels will unavoidably increase the training time. To balance the computational cost and performance, we believed that 3 kernels in a single CB was a reasonable choice.

**Table 6:** Quantitative results (mean) associated with different numbers of convolutional kernels in CBs for combinations of noise levels.

Noise level of testing data		SCN-2	SCN-3	SCN-4
*b*_0_ = 5×10^5^	**PSNR**	45.3385	45.5520	**45.7072**
	**RMSE**	0.0055	0.0054	**0.0053**
	**SSIM**	0.9824	0.9836	**0.9837**
*b*_0_ = 1×10^5^	**PSNR**	42.0861	42.2169	**42.6289**
	**RMSE**	0.0081	0.0080	**0.0076**
	**SSIM**	0.9687	0.9698	**0.9725**
*b*_0_ = 5×10^4^	**PSNR**	40.7229	41.2028	**41.4025**
	**RMSE**	0.0096	0.0090	**0.0088**
	**SSIM**	0.9592	0.9642	**0.9655**

## Conclusions

We proposed a novel network structure, aided by stacked competitive blocks, for LDCT image restoration. The primary advantage of this method is the introduction of multi-scale processing. Based on two public databases, our proposed SCN achieved the best performance compared with other competing methods, in terms of noise suppression and structural preservation. Next, we are planning to optimize SCN ulteriorly and try to apply it to the wider range of medical imaging tasks.
